# Are We Measuring ADHD or Anxiety? Examining the Factor Structure and Discriminant Validity of the Adult ADHD Self-Report Scale in an Adult Anxiety Disorder Population

**DOI:** 10.1177/10731911231225190

**Published:** 2024-01-30

**Authors:** Arij Alarachi, Colleen Merrifield, Karen Rowa, Randi E. McCabe

**Affiliations:** 1 Anxiety Treatment and Research Clinic, St. Joseph’s Healthcare Hamilton, Ontario, Canada; 2 Department of Psychology, Neuroscience, & Behaviour, McMaster University, Hamilton, Ontario, Canada; 3 Psychiatry and Behavioural Neurosciences, McMaster University, Hamilton, Ontario, Canada

**Keywords:** anxiety disorders, attention-deficit/hyperactivity disorder, confirmatory factor analysis, bifactor models, adult populations

## Abstract

Adults with clinical anxiety have significant symptom overlap and above average rates of attention-deficit/hyperactivity disorder (ADHD). Despite this, ADHD remains a vastly under-detected disorder among this population, indicating the need for a screener with well-understood symptom dimensions and good discriminant validity. The current study compared competing models of ADHD as well as discriminant properties of self-reported ADHD symptoms as measured by the Adult ADHD Self-Report Scale (ASRS-v1.1) in 618 adults with clinical anxiety. A three-factor correlated model of Inattention, Impulsivity, and Hyperactivity, with the movement of one item, *talks excessively*, to a factor of Impulsivity from Hyperactivity fit better than the one-factor, two-factor, and traditional three-factor models of ADHD. Discriminant properties of the screener were fair to good against measures of clinical anxiety and distress; however, some items within the Hyperactivity factor (e.g., difficulty relaxing; feeling driven by a motor) loaded more strongly onto factors of clinical anxiety than ADHD when measures were pooled together. These results suggest that clinicians making differential diagnoses between adult ADHD and anxiety or related disorders should look for evidence of ADHD beyond the overlapping symptoms, particularly for those within the Hyperactivity factor.

In contrast to widely held views that attention-deficit/hyperactivity disorder (ADHD) among adults is a rare phenomenon, epidemiological research has reported a global 12-month prevalence rate between 2.5% and 2.8% (Fayyad et al., 2017; Song et al., 2021). In higher-income countries (e.g., the United States, France, and the Netherlands), the prevalence rate of adult ADHD has been reported to be approximately 3.6% (Fayyad et al., 2017). ADHD is a neurodevelopmental disorder that tends to co-occur with other mental disorders, one of the most common being anxiety and related disorders (34.2%; Fayyad et al., 2017). Mechanisms contributing to this overlap are not fully understood; however, some theorists suggest a neurobiological genesis of this comorbidity. For example, the overlap in symptoms between clinical anxiety and ADHD may be explained by shared genetic risk factors (e.g., Carey et al., 2016; Faraone & Larsson, 2019), increasing susceptibility to the development of either or both disorders. Nigg et al. (2004) also suggested that a complex interplay between neurocognitive factors and temperament is implicated in this comorbidity. They postulated that comorbid ADHD and anxiety may develop through two distinct pathways; one, in which children with cognitive impairments experience high levels of anxiety as a side-effect or secondary symptom of their executive function deficits (i.e., poor cognitive-emotional regulation leading to worry; Nigg et al., 2004). The second pathway occurs when children with high levels of anxious or intrusive thoughts experience higher cognitive load (i.e., due to severe anxiety), leading to secondary inattentive symptoms, yet mostly intact executive functioning (Nigg et al., 2004). Later in development, environmental factors such as negative social responses to ADHD symptoms (e.g., conflict with parents and teachers, rejection from peers) and experiences of hardships or challenges during important developmental milestones (e.g., difficulty in school, risky behaviors) may lead individuals to internalize negative feedback and develop negative beliefs that result in the development of anticipatory anxiety and negative emotional states (Bramham et al., 2012; Psychogiou et al., 2007). Research on the etiology of comorbid ADHD and anxiety or related disorders is also complicated by the different presentations of ADHD (e.g., Primarily Inattentive, Primarily Hyperactive/impulsive, or Mixed), and the vast range of anxiety or related disorders (e.g., social anxiety, generalized anxiety, or obsessive-compulsive disorder [OCD], panic disorder [PD]), leading to heterogeneous pathways for development of both disorders.

Although limited in number, studies involving clinical samples of patients with anxiety or related disorders reveal high rates of undetected comorbid ADHD. Van Ameringen and colleagues (2011) examined 129 consecutive outpatient anxiety disorder clinic admissions. They found that 27.9% of adult patients met criteria for ADHD as assessed by the ADHD module of the Mini International Neuropsychiatric Interview (MINI) Plus (Sheehan et al., 1998). Among these patients, only 16.7% had ever received treatment for ADHD, and only 2.8% were currently receiving treatment for ADHD. Similarly, another clinical study of adult patients with a principal diagnosis of generalized anxiety disorder (GAD) found that 32% met criteria for ADHD in childhood, with half of those continuing to present with clinically significant symptoms in adulthood (Safren et al., 2001). Although these studies indicate a high prevalence of ADHD within adult anxiety or related disorder populations, symptom overlap could contribute to misdiagnosis. For example, individuals may present with symptoms consistent with social anxiety disorder (e.g., a patient reports they worry about their manager negatively evaluating them); however, the anxiety may be revealed as secondary to untreated ADHD upon further investigation (e.g., they routinely arrive to work late and have trouble concentrating and remembering their manager’s instructions). Alternatively, individuals may experience ADHD-like symptoms that are genuinely cognitive features of anxiety or related disorders (e.g., poor concentration due to generalized anxiety disorder).

Differential diagnosis between the two disorders may also be influenced by various forces contributing to false positive cases of ADHD. Research by Barkley et al. (2011) suggests that adults who self-refer to ADHD assessment and treatment without a childhood diagnosis tend to have higher levels of anxiety and lower levels of impairment. Specifically, some patients seeking an ADHD diagnosis due to self-identified difficulties with concentration may be driven by unrealistic expectations of their cognitive abilities due to perfectionism or anxiety rather than true deficits in their executive functioning (Barkley et al., 2011; Sibley, 2021). Diagnosis-seeking adults may also be motivated by secondary gains (e.g., seeking accommodations at work or school or use of stimulant medications), leading to non-credible reports of symptoms (e.g., Harrison et al., 2023, Suhr et al., 2008; 2022). Alternatively, patients with experiences of significant stress or trauma may have deficits in executive functioning that mimic ADHD symptoms (Spann et al., 2012; Szymanski et al., 2011).

Research that has paid careful attention to symptom overlap continues to find high prevalence rates of ADHD in individuals with anxiety or related disorders (Pehlivanidis et al., 2014), suggesting that routine screening followed by rigorous assessment can help bypass some of the differential diagnostic challenges. Undetected ADHD in adults with anxiety or related disorders is problematic, as it is linked to poor clinical outcomes including poor treatment adherence (El Alaoui et al., 2015) and deficits in emotion regulation, organization, and problem-solving as compared with those with symptoms of only anxiety disorder or ADHD (Jarrett, 2016). Taken together, the research suggests that patients presenting with concentration difficulties should be routinely screened for both anxiety and ADHD, while those presenting with heavily overlapping symptoms (e.g., a general sense of being overwhelmed, concentration difficulties) should be assessed for both anxiety or related disorders and ADHD, as these symptoms may require additional clinical attention to promote positive treatment outcomes.

The first step toward accurate assessment of ADHD, among other psychiatric disorders, is the administration of a self-report screener. Given increasing burdens on public and private health care systems, limited resources, and long waitlists for specialized care, initial screening is necessary to determine whether comprehensive diagnostic investigation is warranted. A widely used screening instrument is the 18-item Adult Self-Report Scale v1.1 (ASRS-v1.1), a self-report questionnaire of ADHD symptoms developed jointly by the World Health Organization and Kessler et al. (2005). The ASRS-v1.1 has been validated in the general population in various languages, in adolescent populations, and in clinical populations (e.g., Adler et al., 2012; Kessler et al., 2005; Kiatrungrit et al., 2017; Kim et al., 2013). Currently, there are no studies that have examined the psychometric properties of the ASRS-v1.1 in an anxiety or related disorder population, despite the high level of comorbidity and significant symptom overlap. However, there has been research in other clinical samples with high comorbidity rates of ADHD, including populations of individuals with substance use (Daigre et al., 2015; van de Glind et al., 2013), eating disorders (Carlucci et al., 2017), and depressive disorders (Dunlop et al., 2018). In the closest relative sample to anxiety or related disorders, depressive disorders, the ASRS-v1.1 demonstrates fair sensitivity (60%) and adequate specificity (68.6%; Dunlop et al., 2018).

The ASRS-v1.1 represents the *Diagnostic and Statistical Manual of Mental Disorders* (4th ed.; *DSM-IV*; American Psychiatric Association [APA], 1994) diagnostic criteria for ADHD. In both the *DSM-IV* and *Diagnostic and Statistical Manual of Mental Disorders* (5th ed.; *DSM-5*; APA, 2013), the symptoms of ADHD are divided into two distinct factors: Inattention and Hyperactivity/Impulsivity (H/I), with the only differences in the *DSM-5* being a requirement of fewer symptoms among adults than children (APA, 2013), and that symptoms must have emerged by age 12, as opposed to age 7 in *DSM-IV*. However, previous editions of the *DSM* conceptualized the factor structure of the symptoms differently. The *Diagnostic and Statistical Manual of Mental Disorders* (3rd ed.; *DSM-III*; APA, 1980) was the first to introduce a list of specific diagnostic criteria for the diagnosis of ADHD and divided the symptoms into three distinct factors: Inattention, Hyperactivity, and Impulsivity. The *Diagnostic and Statistical Manual of Mental Disorders* (3rd ed., rev.; *DSM-III-R*; APA, 1987) reflected a theoretical shift to conceptualizing ADHD as having one underlying factor and clustered all symptoms of ADHD together.

Multiple models reflecting these diagnostic changes in the clustering of ADHD symptoms have been put forth, yet research testing these models using the ASRS-v1.1 has been sparse. Moreover, no studies have investigated the factor structure of the measure in anxiety or related disorder populations. Of the few existing studies on factor structure of the ASRS-v1.1, findings are mixed. There is some support for a two-factor structure for the ASRS-v1.1 in adult populations, consistent with the *DSM-5* (Hesse, 2013; Lozano et al., 2020). However, these studies examined only the six-item screening version of the scale (ASRS-6), limiting the interpretation of these results. Another study found converging evidence for two factors of Inattention and H/I using the full 18-item ASRS-v1.1 in a large sample of both adults with ADHD and population controls (Brevik et al., 2020). There is also overwhelming support for the three-factor model of ADHD (Knouse et al., 2009; Kooij et al., 2005; Park et al., 2018; Span et al., 2002) for both bifactor and first-order structures, consistent with both *DSM-III* and -*III-R* conceptualizations. In a bifactor model, items load onto a general ADHD factor but also a respective specific factor with all factors orthogonal to one another. However, some researchers argue that even though statistical fit appears to be comparable or better in these bifactor models, first-order models contain fewer parameters and, thereby, are the most parsimonious and theoretically valid (Gomez & Stavropoulos, 2021; Park et al., 2018).

Evidence from more recent research using other measures diverges from traditionally defined *DSM* factors. Gibbins and colleagues (2012) examined symptom structure in 751 adults with ADHD using the ADHD Rating Scale–Fourth Edition (ADHD-RS-IV; DuPaul et al., 1998) and found that one item traditionally considered a symptom of hyperactivity, *talks excessively*, loaded better onto the Impulsivity factor, leading to a regrouping of items within these domains. They renamed the specific factors Inattention, Motor H/I (i.e., squirming/fidgeting, feeling driven by a motor, leaving one’s seat, feeling restless, and having difficulty relaxing/unwinding), and Verbal H/I (i.e., excessive talking, finishing other’s sentences, difficulty waiting for one’s turn, and interrupting others; Gibbins et al., 2012). This structure was further supported in a study on a large psychiatric outpatient group of 1,094 adults using the ASRS-v1.1 (Stanton et al., 2018). However, this reconceptualization of factors may lack content validity, given the differences in the two constructs of Hyperactivity (e.g., excessive motor activity or urges to engage in activity) and Impulsivity (e.g., problems with refraining from engaging in behavior that may be unhelpful within a particular situation). Moreover, phenomenological appropriateness of item fit within each factor should be examined, as *driven by a motor*, may manifest as both verbal and motoric in nature, and *restless or fidgety*, may be interpreted by respondents as mental restlessness and not meet criteria for Verbal H/I or Motor H/I. Nonetheless, other studies with child populations have found similarly improved fit for the three-factor model when the *talks excessively* item was moved from Hyperactivity to Impulsivity (Arildskov et al., 2022; Gomez et al., 2021), as is conceptualized in the International Classification of Diseases-10 (ICD-10; World Health Organization, 1993).

Taken together, previous research indicates discrepancies in both the optimal number of factors (one, two, or three) and the structuring of items within factors (e.g., with *talks excessively* under hyperactivity vs. impulsivity) in adult populations with ADHD and in general psychiatric populations. Thus, further investigation is necessary to reconcile the mixed findings on the factor structure of this often-used instrument in samples of adults with anxiety or related disorders, given the high rates of comorbidity between anxiety disorders and ADHD.

Moreover, due to the significant overlap in symptoms of ADHD and anxiety or related disorders, the high prevalence of this comorbidity, and the dearth of research examining the ASRS-v1.1’s performance in clinical anxiety populations, it is essential to examine the discriminant validity of the measure. It is necessary to ensure the ASRS-v1.1 is not erroneously capturing anxiety symptoms or general distress in this population.

As such, the purpose of the present study was to assess the factor structure of the ASRS-v1.1 with data from a large Canadian clinical sample of adults presenting with a principal diagnosis of an anxiety or related disorder. This study explored three main research questions including (a), is the ASRS-v1.1 a structurally valid tool to screen for ADHD in an anxiety or related disorders sample; (b), which model fits best, in terms of the number of factors and factor types (i.e., traditional *DSM* factors or novel motoric and verbal split of hyperactivity and impulsivity); (c) does the ASRS-v1.1 demonstrate adequate discriminant validity within this population, or are there items that are contaminated by symptoms of clinical anxiety or distress?

## Method

### Participants

Participants were 619 adults seeking treatment at a tertiary care outpatient anxiety clinic located in an academic teaching hospital. Services at the clinic are fully funded by the government health plan and thus accessible to anyone in the community. To qualify for the study, participants were required to have a principal diagnosis of PD (with or without agoraphobia), social anxiety disorder, GAD, or OCD. Diagnostic criteria were based on the *DSM-5* (5th edition, APA, 2013).

### Measures

#### ADHD Measure

The ASRS-v1.1 is composed of 18 questions based on *DSM-IV* criteria for ADHD. Respondents rate the frequency of symptoms on a 5-point Likert-type scale (0 = *never*, 1 = *rarely*, 2 = *sometimes*, 3 = *often*, 4 = *very often*). Questions were developed specifically for adult respondents, rather than children, using language that places symptoms in a context to which adults can relate. For example, the wording for the question assessing the *DSM-IV* symptom “often loses things necessary for tasks or activities” was changed to “how often do you misplace or have difficulty findings things at home or work?” The ASRS-v1.1’s psychometric properties rely heavily on the number of items, scoring method, and population it is being used. In the initial validation of the ASRS-v1.1 with a general population sample, Kessler et al. found excellent total classification accuracy (96.2%) and overall good discriminant validity, with adequate specificity (56.3%) and excellent sensitivity (98.3%; 2005). When scored differently (e.g., based on *DSM-5* criteria), the ASRS-v1.1 demonstrated strong sensitivity (91%) and acceptable specificity (74%; Ustun et al., 2017). In some comorbid clinical populations (e.g., substance use disorders), discriminant validity appears to be more robust, while in other clinical populations with more overlapping symptoms (e.g., depressive disorders), discriminant validity is weaker. The ASRS-v1.1 was chosen given its widespread use, simplicity (e.g., does not require clinicians to reference norms), and cost-effectiveness (e.g., free of charge). The ASRS-v1.1 can be obtained free of charge online at the following website: http://www.hcp.med.harvard.edu/ncs/ftpdir/adhd/6Question-ADHD-ASRS-v1-1.pdf.

#### Anxiety or Related Disorder Measures

To measure anxiety or related disorder-specific symptoms, four measures based on the principal diagnosis at the time of the assessment were used. These included a brief seven-item measure of GAD, the Generalized Anxiety Disorder-7 Item Scale (GAD-7; Spitzer et al., 2006); an 18-item measure of the severity of OCD symptoms, the Obsessive-Compulsive Inventory-Revised (OCI-R; Foa et al., 2002); a 17-item measure of the severity of panic attacks and disorder, the Panic Disorder Severity Scale–Self Report (PDSS-SR; Shear et al., 1997) and a 17-item measure of social anxiety severity, the Social Phobia Inventory (SPIN; Connor et al., 2000). For all measures, respondents were asked to rate the frequency of symptoms in the past 1 or 2 weeks on a Likert-type scale.

#### Distress Measure

To measure general distress levels, participants completed the Depression Anxiety and Stress Scale 21 (DASS-21; Lovibond & Lovibond, 1995). This 21-item measure consists of three subscales with seven items each, measuring symptoms of depression, anxiety, and stress. For all symptoms, participants were asked to rate frequency of symptoms in the past week on a Likert-type scale, with zero corresponding to the response of, *did not apply to me at all*, and four corresponding to, *applied to me very much or most of the time*. Research has found good internal consistency and concurrent validity for the measure (Antony et al., 1998).

### Procedures

Data were collected as part of the clinic’s standard intake process. All participants provided written informed consent to participate in research and study procedures were approved by the institution’s Research Ethics Board. This study was not preregistered. Participants were assessed through a psychiatric consultation, nursing intake appointment, or by a clinician using the *Diagnostic Assessment Research Tool* (DART; McCabe et al., 2017). The DART is an open access semi-structured interview that supports diagnostic assessment of various psychological disorders, with strong initial psychometric validation (Schneider et al., 2022). The DART interviews comprise a package of “core modules” that assessed for a range of anxiety; mood; obsessive-compulsive and related; trauma- and stressor-related; and substance-related and addictive disorders, relevant to the clinical population. Additional optional modules (e.g., insomnia disorder, depersonalization/derealization disorder) were used to assess for other mental health disorders, depending on the pertinence to the referral question and patients’ symptoms. Comprehensive assessment and diagnosis of ADHD was not conducted as part of the routine DART interview process. Instead, clinicians assessed for symptoms using an optional ADHD symptom screening module and when necessary, referred patients to a psychiatry consultation, primary care physician, or a neuropsychological clinic for further investigation. Moreover, information on previous diagnoses (e.g., ADHD) was collected during the DART and recorded for clinical and research purposes, though ADHD in childhood was not specifically queried. The principal diagnosis was based upon the disorder that was found to be most disabling at the time of the assessment. DART interviewers included psychologists, post-doctoral fellows in clinical psychology, social workers, and graduate-level clinical psychology practicum students, all of whom received extensive training and supervision in conducting the interview. Psychiatric consultations may have involved more comprehensive ADHD assessment and diagnosis, depending on the relevance to the referral query. As part of the clinic intake process, all participants completed a package of self-report questionnaires that included the ASRS-v1.1 (i.e., including those without suspected ADHD), disorder-specific symptom severity questionnaires based on their principal diagnosis, and the DASS-21.

### Data Analyses

#### ADHD Criterion A

Given that ADHD was not assessed in our sample, the rate of participants who likely met *DSM-5* Criterion A for the diagnosis based on the ASRS-v.1.1 was derived. ASRS-v1.1 items were coded as present if respondents endorsed they occurred “often,” following the *DSM-5* diagnostic criteria. Following the recoding, participants with five or more Inattentive and/or Hyperactive/Impulsive symptoms were coded as meeting Criterion A for ADHD.

#### Models

A first-order one-factor model proposes that all 18 items load onto a single factor, implying a unitary construct. This model is consistent with the *DSM-III-R* model of ADHD. A two-factor model proposes that the nine items describing inattentive symptoms load onto an Inattention factor, while the six items describing hyperactive and three items describing impulsive symptoms load onto one H/I factor. This model is consistent with the *DSM-IV* and *DSM-5* models of ADHD. A first-order three-factor model specifies that each of the 18 items loads onto one of three underlying factors, whereby nine items describing inattentive symptoms load onto an Inattention factor, six items describing hyperactive symptoms load onto a factor of Hyperactivity, and three items describing impulsive symptoms load onto a factor of Impulsivity. This model is consistent with the *DSM-III* model for ADHD. Finally, the movement of one item *talks excessively*, from Hyperactivity to Impulsivity, specifies a revised three-factor model with nine items describing inattentive symptoms loading onto an Inattention factor, four items describing impulsive symptoms loading onto a revised factor of Impulsivity (denoted Impulsivity-*r*), and five items describing hyperactive symptoms loading onto a revised factor of Hyperactivity (denoted Hyperactivity-r). Although this model has not been put forth by the *DSM*, previous studies have found evidence for this factor structure in adult populations (Gibbins et al., 2012; Stanton et al., 2018). This model was considered in the current study to further investigate its utility. Higher-order models were not included in analyses as they would be under-identified or just-identified (e.g., Rindskopf & Rose, 1988), precluding their suitability for testing goodness of fit. A model is considered suitable to test goodness of fit when both the measurement model for items and the structural model for factors are overidentified (e.g., meaning the difference between known values and free parameters is greater than or equal to one).

#### Sample Size

The sample size was deemed acceptable according to established guidelines for meeting confirmatory factor analysis (CFA) sample requirements, which include evaluating the ratio of variables to factors and degree of communality (Mundfrom et al., 2005). Our data had a variable to factor ratio ranging from four to nine depending on the model and demonstrated a wide communality pattern. Based on this, the necessary minimum sample size is estimated to be 350; the current study’s sample size of 619 surpassed this.

#### Confirmatory Factor Analysis

CFA, using Analysis of Moment Structures (AMOS, Version 26.0; Arbuckle, 2019), was utilized to test four potential models underlying the 18 ADHD symptoms. The covariance matrices for responses to the 18 items were used as input and parameters were estimated using maximum likelihood. To ensure identification of the models, one path for each latent variable was constrained to a value of one.

Each model’s fit with the data was evaluated with several goodness-of-fit indices (recommended cut-off values are in parentheses), including the relative chi-square statistic (χ^2^/df; <3; Kline, 1998), the comparative fit index (CFI; >.90 acceptable, >.95 excellent; Bentler, 1990), the Tucker–Lewis index (TLI; >.90 acceptable, >.95 excellent; Tucker & Lewis, 1973), the root mean square error of approximation (RMSEA; <.08 acceptable, <.05 excellent; Browne & Cudeck, 1993), and the standardized root mean square residual (SRMSR; <.08; Hu & Bentler, 1999). Comparisons of fit among models were made using these indices, as well as the Akaike information criterion (AIC; Akaike, 1987), wherein smaller AIC values indicate better fit (Hu & Bentler, 1995) and a difference of ≥10 between two models is considered meaningful (Burnham & Anderson, 1998).

#### Anxiety, Distress, and ADHD Symptoms

Following confirmatory factor analysis, the relationship between disorder-specific anxiety symptoms and ADHD symptoms was examined. Individual Pearson correlational analyses between ADHD and each anxiety or related disorder were conducted, due to the vast differences in clinical symptoms across the four anxiety and related disorders (i.e., generalized anxiety disorder, social anxiety disorder, PD, and OCD). For the best-fitting model, the correlation between specific ADHD factors and anxiety symptoms was derived. Correlations between distress subscale scores as measured by the DASS-21 (stress, anxiety, and depression) and ADHD total scores and specific factors were also derived, giving a total of 28 separate correlational analyses. Given these tests were conducted with the aim of examining the discriminant properties of the ASRS-v.1.1 against measures of clinical anxiety and distress, reducing the risk of Type II error was deemed more critical than risk of Type I overinflation from multiple comparisons. As such, analyses were conducted as usual with alpha = .05. Following this, a *post hoc* Holms–Bonferroni correction was applied for multiple comparisons (Holm, 1979) to report both corrected and uncorrected *p-*values.

To examine the factor structure of combined anxiety and ADHD measures, four separate exploratory factor analysis (EFA) were conducted with pooled ASRS-v1.1 and disorder-specific anxiety measure items using R (v.4.3.0; R Core Team, 2023) with the lavaan package (.6-15; Rosseel, 2012). It was expected that items in these well-validated measures would load relatively well onto their respective factors; thus all EFA models were set up with two factors representing ADHD and anxiety domains *a priori* to determine whether specific items cluster better around their respective factors, the opposite factor, or cross-load.

## Results

### Demographic and Clinical Characteristics

The sample was 66% female with mean age of 32.7 years (*SD* = 11.9 years). Most of the participants had attended some or completed post-secondary education (66.6%). Participants were able to report multiple racial/ethnic identities. Most described themselves as Caucasian/White (84.3%) with the remaining 15.7% describing themselves as Indigenous (6.2%), Black/African/Caribbean (9.3%), Hispanic/Latin (3.1%), East/South/South-East Asian (19.6%), Biracial (13.4%), or other (13.4%). Most participants reported being either single (39.5%) or married (32.2%). Regarding principal diagnosis, 42.3% were diagnosed with generalized anxiety disorder, 26.8% with social anxiety disorder, 18.9 % with OCD, and 12% with PD. The most common comorbid diagnoses were major or persistent depressive disorder (39%), agoraphobia (11%), cannabis use or alcohol use disorder (7.5%), and posttraumatic stress disorder (6.1%). Forty-two percent of the sample had two or more comorbid diagnoses. Twenty-one participants had an additional or past diagnosis of ADHD documented by clinicians in the clinic database, giving a prevalence rate of 3.4% which is likely a gross underrepresentation of true prevalence of ADHD in this sample. Forty-one percent (41.3%) of the sample met *DSM-5* criterion A for ADHD based on ASRS-v.1.1 reported frequencies of each symptom, with 25.5% meeting threshold for a primarily inattentive presentation, and 15.8% meeting threshold for a combined presentation. No individuals met threshold for a primarily hyperactive/impulsive presentation.

### ASRS-v1.1 Descriptive Statistics

The sample’s mean score on the six-item screener of the ASRS-v1.1 was 13.9 (*SD* = 4.2), just below the established clinical threshold of 14 (Ustun et al., 2017). The mean score on the full 18-item ASRS-v1.1 was 38.5 (*SD* = 10.9), scoring within the 95^th^ percentile range of the general population (Kessler et al., 2005). The mean scores for the Inattention and HI factors were 20.68 (*SD* = 6.46) and 17.81 (*SD* = 6.13), respectively. Means and standard deviations for responses to the individual items are provided in Table 1. Given that true ADHD cases were not captured in this sample, determining the distribution of ASRS-v1.1 scores in the sample was deemed essential. A Shapiro–Wilk test was performed and demonstrated that the total ASRS-v1.1 scores were distributed normally (*W* = .997, *p* = .24). This result was also confirmed with visual examination of the histogram of total ASRS-v1.1 score and the QQ plot. However, the Shapiro-Wilk test did not demonstrate normal distribution for specific factors of Inattention (*W* = .993, *p* = .007), or HI (*W* = .995, *p* = .033). Visual examination of the histograms demonstrated a slightly negative skew for Inattention and a slightly positive skew for HI. These results are to be expected in the study’s clinical population and the large sample size; thus, all analyses were conducted as usual.

**Table 1.** Mean and Standard Deviations for the 18 ASRS-v1.1 Items (N = 618).

Item number	Symptom	*M*	*SD*
ASRS1	Difficulty finishing projects	2.12	1.22
ASRS2	Difficulty organizing tasks	2.04	1.10
ASRS3	Problems remembering appointments	2.03	1.19
ASRS4	Delaying tasks	2.81	1.04
ASRS5	Squirming/fidgeting	2.97	1.11
ASRS6	Feeling driven by a motor	1.91	1.16
ASRS7	Careless mistakes	1.84	1.00
ASRS8	Inattention during boring/repetitive tasks	2.57	1.05
ASRS9	Difficulty concentrating when spoken to	2.31	1.10
ASRS10	Misplacing things	2.30	1.13
ASRS11	Distraction by activity/noise	2.66	1.07
ASRS12	Leaving seat	1.12	1.08
ASRS13	Feeling restless/fidgety	2.57	1.05
ASRS14	Difficulty relaxing/unwinding	2.59	1.11
ASRS15	Excessive talking	1.82	1.26
ASRS16	Finishing other’s sentences	1.79	1.25
ASRS17	Difficulty waiting turn	1.44	1.19
ASRS18	Interrupting others	1.58	1.01

*Note.* ASRS = ADHD Self-Report Scale.

### Confirmatory Factor Models

All models were left unadjusted. Fit indices for all models are shown in Table 2. Results indicated that fit was poor for the one-, two-, and three-factor *DSM*-defined models, with unacceptable fit indices (CFI, TLI <.90; RMSEA >.90). The three-factor model of Inattention, Motor H/I, and Verbal H/I demonstrated improved fit on most indices, except for two (CFI, TLI <.90).

**Table 2.** Measures of Fit for Models of ASRS-v1.1 Items.

Model	χ^2^ (*df*)	CFI	TLI	RSMEA	SRMSR	AIC
Confirmatory factor analyses						
One-factor (ADHD)	1,264.89 (135)	.66	.61	.12	.093	1,136.89
Two-factor (inattention and hyper/impulsivity)	952.41 (134)	.75	.72	.10	.078	1,026.41
Three-factor (inattention, hyper, impulsivity)	682.44 (132)	.83	.81	.08	.075	760.44
**Three-factor (inattention, hyper-*r*, impulsivity-*r*)**	**546.21 (132)**	**.88**	**.86**	**.07**	**.063**	**624.68**

*Note. N =* 619. The best-fitting model is shown in boldface. χ^2^ = chi-square statistic; *df* = degrees of freedom; CFI = comparative fit index; TLI = Tucker–Lewis index; RSMEA = root mean square error of approximation; AIC = Akaike information criterion; ADHD = attention-deficit/hyperactivity disorder.

**Figure 1.**
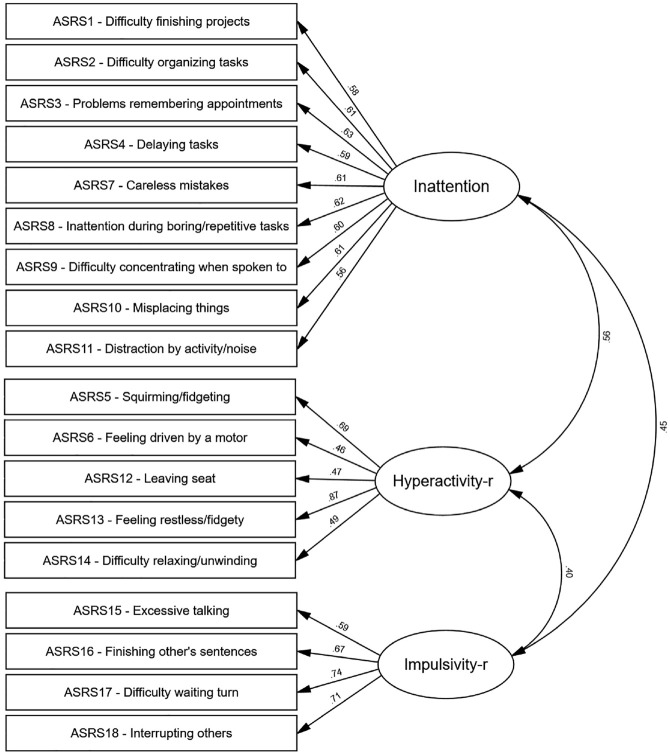
First-Order Correlated Model of ASRS-v1.1 Items With Specific Factors of Inattention, Hyperactivity-r, and Impulsivity-r. *Note.* All factor loadings shown were significant (*p* < .001). ASRS = ADHD Self-Report Scale.

The three-factor model of Inattention, Impulsivity-*r*, and Hyperactivity-*r* shown in Figure 1 represented the best-fitting model of all first-order models, χ^2^/*df* = 4.14; CFI = .88; TLI = .86; RMSEA = .07, SRMR = .063. This observation is further supported by the smaller AIC value observed for this model and a difference >10 in AIC values between it and the other first-order models examined, including the traditional three-factor model of Inattention, Hyperactivity, and Impulsivity (factor structure and loadings for this model can be found in Supplemental Figure S2).

Standardized factor loadings are shown in Supplemental Table S1 and Figure 1. All factor loadings were significant (*p*<.05), with standardized factor loadings ranging from .47 to .87 (Figure 1). Factor loadings were highly similar to the traditional three-factor model (.01–.03 difference in loadings) except for the item *talks excessively*, which demonstrated improved loading when moved from the Impulsivity factor to the Hyperactivity factor (.16 increase). Moreover, average factor loadings were strong for all three specific factors of Inattention (*M* = .6), Hyperactivity-*r* (*M* = .6), and Impulsivity-*r* (*M* = .68) and all three specific factors were moderately correlated with one another. Item to item correlations can be found in Supplemental Table S2.

### Discriminant Validity of ASRS-v1.1 Against Measures of Distress and Clinical Anxiety

#### Correlations Between ASRS-v1.1 and Anxiety and Distress Measures

Results of the correlational analyses are shown in Table 3. ASRS-v1.1 total scores were moderately positively correlated with GAD-7 scores, *r*(260) = .53, *p* < .001, and OCI-R scores, *r*(117) = .45, *p* < .001. Specific factors of Inattention, Motor H/I, and Verbal H/I in the final model demonstrated a moderate correlation in the positive direction with both GAD-7 scores and OCI-R scores.

**Table 3.** Pearson Correlations Between ASRS-v1.1 Total and Specific Factors, Anxiety Measures, and DASS-21 Subscales Scores.

Measure	ASRS-v1.1 total score	Inattention	Hyperactivity-*r*	Impulsivity-*r*
GAD-7 (*N* = 119; *M* = 12.92; *SD =* 4.84)	.53* (.38, .65)	.37* (.21, .52)	.54* (.4, .66)	.31* (.14, .46)
OCI-*R* (*N* = 117; *M* = 25.57; *SD =* 14.08)	.45* (.28, .58)	.37* (.19, .52)	.41* (.24, .56)	.27* (.09, .44)
PDSS (*N* = 72; *M* = 14.58; *SD =* 6.12)	.34* (.12, .53)	.36* (.14, .55)	**.3** (.08, .5)	.08 (-.16, .31)
SPIN (*N* = 161; *M* = 46.5; *SD =* 10.84)	.11 (-.04, .26)	.08 (-.07, .26)	.15 (-.01, .3)	.03 (-.13, .18)
DASS-21 Anxiety (*N* = 614; *M* = 18.14; *SD =* 9.53)	.4* (.33, .46)	.33* (.26, .4)	.4* (.33, .46)	.19* (.12, .27)
DASS-21 Stress (*N* = 614; *M* = 22.25; *SD =* 9.06)	.56* (.51, .62)	.43* (.36, .49)	.54* (.49, .6)	.37* (.3, .43)
DASS-21 Depression (*N* = 614; *M* = 19.7; *SD =* 11.21)	.39* (.32, .45)	.42* (.35, .48)	.3* (.23, .37)	**.1** (.02, .18)

*Note.* Values with boldface indicate significance at *p* < .05. Values with boldface and an asterisk indicate significance is maintained following the Holm–Bonferroni correction for multiple comparisons. Only the significance of correlations between PDSS and Hyperactivity-*r* and DASS-21 and Impulsivity-*r* are lost following the correction. ASRS = ADHD Self-Report Scale; DASS-21 = Depression Anxiety and Stress Scale 21; GAD-7 = Generalized Anxiety Disorder-7; OCI-R = Obsessive-Compulsive Inventory-Revised; PDSS = Panic Disorder Severity Scale; SPIN = Social Phobia Inventory.

ASRS-v1.1 total scores were also found to be moderately correlated with PDSS scores, *r*(70) = .34, *p* = .003. There were also moderate, positive, and significant correlations between specific factors of Inattention and Motor H/I (without the Holms–Bonferroni correction). The relationship between ASRS-v1.1 total scores and SPIN scores were weakly correlated, and non-significant *r*(164) = .11, *p* = .16. Similarly, all correlations between the specific factors and the SPIN scores were weak and non-significant.

With regards to distress, the ASRS-v.1.1 total scores were moderately correlated with each subscale of Anxiety, Stress, and Depression on the DASS-21. The largest correlations were between ASRS-v1.1 total scores and DASS-21 Stress subscale, *r*(614) = .56, *p* < .001 and the specific factor of Motor H/I and DASS-21 Stress subscale *r*(614) = .54, *p* < .001. There were also low to moderate positive correlations between each subscale and each specific ADHD factor.

#### EFA of Item Pools

Overall, items mostly loaded onto their appropriate factors (i.e., ADHD or anxiety or related disorder), with some items in the pooled ADHD-GAD and pooled ADHD-OCD models demonstrating better fit onto the alternative factor or cross-loading. Cut-off values for factor loadings were set to .30 and significance level was set to *p* < .01.

##### GAD and ADHD

Loadings for the EFA item pool with GAD and ADHD measures are shown in Table 4. All ASRS-v1.1 items except for Item 3, *problems remembering appointments;* 14, *difficulty unwinding;* and 15, *talking too much in social situations*, demonstrated acceptable, significant loadings onto the ADHD factor, with loadings ranging from .35 to .71. ASRS-v1.1 Item 14 loaded significantly onto the GAD factor (.49). All GAD-7 items except for Item 6, *becoming easily irritable and annoyed*, demonstrated acceptable and significant loadings onto the generalized anxiety factor, with loadings ranging from .32 to .91. GAD-7 Items 5, *being so restless that it is hard to sit still*, and 6 also demonstrated a significant loading onto the ADHD factor (.48 and .40, respectively).

**Table 4.** Standardized Factor Loadings for Pooled ASRS-v1.1 and GAD-7 Items.

Item name	Item description	Factor 1 (ADHD)	Factor 2 (GAD)
ASRS1	Difficulty finishing projects	.45*	−0.13
ASRS2	Difficulty organizing tasks	.44*	−.01
ASRS3	Problems remembering appointments	.29*	.12
ASRS4	Delaying tasks	.35*	.13
ASRS5	Squirming/fidgeting	.52*	.11
ASRS6	Feeling driven by a motor	.42*	−.05
ASRS7	Careless mistakes	.72*	−.13
ASRS8	Inattention during boring/repetitive tasks	.55*	.01
ASRS9	Difficulty concentrating when spoken to	.58*	.07
ASRS10	Misplacing things	.52*	−.09
ASRS11	Distraction by activity/noise	.55*	−.06
ASRS12	Leaving seat	.56*	.08
ASRS13	Feeling restless/fidgety	.56*	.08
ASRS14	Difficulty relaxing/unwinding	.01	.49*
ASRS15	Excessive talking	.22	.08
ASRS16	Finishing other’s sentences	.48*	−.09
ASRS17	Difficulty waiting turn	.53*	.02
ASRS18	Interrupting others	.49*	−.004
GAD7-1	Feeling nervous, anxious, or on edge	.01	.89*
GAD7-2	Not being able to stop or control worrying	−.04	.91*
GAD7-3	Worrying too much about different things	−.004	.90*
GAD7-4	Trouble relaxing	.06	.65*
GAD7-5	So restless that it is hard to sit still	.48*	.32*
GAD7-6	Becoming easily annoyed or irritable	.40*	.21
GAD7-7	Feeling afraid	.11	.56*

*Note.* Values with an asterisk indicate significance at *p* < .01 and in boldface indicate a stable factor loading (e.g., at least .|30|). ASRS = ADHD Self-Report Scale; GAD-7 = Generalized Anxiety Disorder-7; ADHD = attention-deficit/hyperactivity disorder.

##### OCD and ADHD

Loadings for the EFA item pool with OCD and ADHD measures are shown in Table 5. All ASRS-v1.1 items except for Item 6, *driven by a motor;* 14, *difficulty unwinding;* and 16, *finishing the sentences of others*, demonstrated acceptable, significant loadings onto the ADHD factor, with loadings ranging from .40 to .73. ASRS-v1.1 Item 6 loaded significantly onto the OCD factor (.35). Several OCI-R items demonstrated stronger and significant loadings onto the ADHD factor, particularly for Items 7 and 13, which correspond to the hoarding subscale, and Items 6 and 12, which correspond to the obsessing subscale.

**Table 5.** Standardized Factor Loadings for Pooled ASRS-v1.1 and OCI-R Items.

Item name	Item description	Factor 1 (ADHD)	Factor 2 (OCD)
ASRS1	Difficulty finishing projects	.66*	−0.15
ASRS2	Difficulty organizing tasks	.63*	−.23
ASRS3	Problems remembering appointments	.73*	−.14
ASRS4	Delaying tasks	.53*	.09
ASRS5	Squirming/fidgeting	.49*	.03
ASRS6	Feeling driven by a motor	.12	.35*
ASRS7	Careless mistakes	.52*	−.24
ASRS8	Inattention during boring/repetitive tasks	.62*	−.10
ASRS9	Difficulty concentrating when spoken to	.63*	.02
ASRS10	Misplacing things	.61*	−.19
ASRS11	Distraction by activity/noise	.42*	.23
ASRS12	Leaving seat	.42*	.02
ASRS13	Feeling restless/fidgety	.58*	.09
ASRS14	Difficulty relaxing/unwinding	.27*	.14
ASRS15	Excessive talking	.54	.05
ASRS16	Finishing other’s sentences	.29*	.15
ASRS17	Difficulty waiting turn	.41*	.14
ASRS18	Interrupting others	.46*	.02
OCI-R1	Saving up so many things	.40*	.12
OCI-R2	Checking things often	.26*	.44*
OCI-R3	Upset if objects are not arranged properly	−.03	.90*
OCI-R4	Feeling compelled to count	.18	.43*
OCI-R5	Difficulty touching objects	.07	.36*
OCI-R6	Difficulty controlling thoughts	.45*	.05
OCI-R7	Collecting things that are not needed	.42*	.25*
OCI-R8	Repeated checking of doors, windows	.19	.46*
OCI-R9	Upset if others change arrangements	.002	.87*
OCI-R10	Have to repeat certain numbers	.30*	.30*
OCI-R11	Cleaning because of feeling contaminated	.01	.34*
OCI-R12	Upset by intrusive unpleasant thoughts	.42*	–.01
OCI-R13	Avoid throwing things away	.43*	.23
OCI-R14	Repeatedly checking gas, water taps	.21	.42*
OCI-R15	Need things to be arranged in certain way	−.004	.92*
OCI-R16	Good and bad numbers	.34*	.29*
OCI-R17	Frequent and unnecessary handwashing	−.13	.26*
OCI-R18	Frequent nasty thoughts	.26*	.05

*Note.* Values with an asterisk indicate significance at *p* < .01 and in boldface indicate a stable factor loading (e.g., at least .|30|). ASRS = ADHD Self-Report Scale; OCI-R = Obsessive-Compulsive Inventory-Revised; ADHD = attention-deficit/hyperactivity disorder; OCD = obsessive-compulsive disorder.

##### PDSS and ADHD

All but one ASRS-v1.1 items loaded onto their respective ADHD factor significantly, with loadings ranging from .38 to .80. Item 6, *driven by a motor*, demonstrated a poor, nonsignificant loading onto both the ADHD factor (.21) and the PD factor (.02). All PDSS items were significant and strongly loaded onto their respective factor, with loadings ranging from .56 to .81.

##### SAD and ADHD

All ASRS-v1.1 items loaded onto their respective ADHD factor significantly, with loadings ranging from .31 to .65. All SPIN items loaded onto their respective social anxiety factor significantly, with loadings ranging from .43 to .71. Item 9 on the SPIN, *avoiding activities where I am the center of attention*, had a negative significant loading onto the ADHD factor. No other items demonstrated cross-loadings.

## Discussion

The overall aim of this study was to examine the structural and discriminant validity of ASRS-v1.1-measured ADHD symptoms in a large sample of adults with a principal anxiety or related disorder. We found the strongest support for the three-factor model of ADHD; Inattention, Impulsivity-*r*, and Hyperactivity-*r*, in which the item *talks excessively* was moved from the factor of Hyperactivity to Impulsivity. The ASRS-v1.1 demonstrated only fair discriminant validity against measures of GAD, OCD, and a measure of distress. Discriminant validity of the Impulsivity factor was the strongest of all three ADHD factors, whereas that for the Hyperactivity factor was the poorest. Some specific items within the Hyperactivity factor showed poor discrimination against anxiety measures, warranting consideration and perhaps concern with their contribution to screening positive for ADHD.

### Factor Structure

The superiority in the fit of the three-factor model over the two-factor and the one-factor model of ADHD in this study is consistent with *DSM-III* and ICD conceptualizations of ADHD symptom domains. Previous research in adult populations with confirmed diagnoses of ADHD, other psychiatric disorders, and community samples have also found support for the separation of hyperactivity and impulsivity into separate factors (Callahan & Plamondon, 2019; Gibbins et al., 2012; Gomez & Stavropoulos, 2021; Knouse et al., 2009; Kooij et al., 2005; Morin et al., 2016; Park et al., 2018; Proctor & Prevatt, 2009; Span et al., 2002; Stanton et al., 2018). The superiority of the three-factor model in our study suggests Impulsivity and Hyperactivity are distinct constructs in adult psychiatric populations with comorbid symptoms of ADHD, each warranting separate consideration for ADHD screening and assessment. Span et al. (2002) suggest that Impulsivity may emerge as a distinct construct for self-rating adult populations who have more developed improved self-monitoring abilities than children who may conflate symptoms of hyperactivity and impulsivity. Within an anxiety or related disorders population, it may be particularly important to parse out items within each factor, given the differences in their overlap (and potential for misdiagnosis) with clinical anxiety symptoms. For example, we found that the items within the Hyperactivity factor showed the weakest discriminant properties against measures of anxiety and distress, whereas the Impulsivity factor was the best at discriminating against anxiety and distress (discussed further in sections below).

Loadings for the best-fitting three-factor model were slightly lower than in other studies (e.g., approximately .05–.2 difference between loadings in the current study and loadings in studies by Glutting et al., 2005; Park et al., 2018; Proctor & Prevatt, 2009). This discrepancy may be due to differences in the diagnostic makeup of the samples between the current study and previously used samples in other studies. All participants in the current study had a principal diagnosis of an anxiety or related disorder and only approximately three percent were diagnosed with ADHD (although 41% had probable ADHD, as determined by the symptom count for the ASRS-v.1.1 for items endorsed as “often,” consistent with the *DSM-5*-TR Criterion A). In other studies, the samples consisted of college students and parents of children with ADHD who had higher confirmed rates of ADHD (Glutting et al., 2005; Park et al., 2018; Proctor & Prevatt, 2009). Higher levels of noise due to substantial symptom overlap with clinical anxiety and general distress may have attenuated the capacity of ADHD factors to explain variance as well as they do in other studies with higher rates of confirmed ADHD. Nonetheless, the convergence of the three-factor structure in the current study with the structure found in community samples, adults with confirmed diagnoses of ADHD, and broader psychiatric populations suggests that the ASRS-v1.1 appears to be a structurally valid tool in an anxiety or related disorders population. Further investigation of the psychometric properties of the ASRS-v1.1 in this clinical population is essential to corroborate the validity of this tool as a useful screening measure.

The findings of this study add to a growing body of work suggesting that the symptom *excessive talking* is better captured within an Impulsivity factor rather than within a Hyperactivity factor (Gibbins et al., 2012; Glutting et al., 2015; Morin et al., 2016; Stanton et al., 2018). Some researchers have even renamed the distinct factors Motor H/I (e.g., representing motor or physical manifestations of symptoms) and Verbal H/I (e.g., representing verbal or communicative manifestations of symptoms; Gibbins et al., 2012; Stanton et al., 2018). However, phenomenological appropriateness of item fit within these reconceptualized factors should be considered, as Item 6, *driven by a motor*, may manifest as both verbal and motoric in nature, and Item 13, *restless or fidgety*, may be interpreted by respondents as mental restlessness and not meet criteria for either Verbal H/I or Motor H/I. Instead, its plausible that the item *talks excessively* is simply a symptom of Impulsivity as is conceptualized in the ICD-10 (Glutting et al., 2005; Gomez et al., 2021; Morin et al., 2016). This reconceptualization may be particularly advantageous when screening for ADHD in clinical anxiety populations, in which excessive levels of response inhibition (i.e., a component of impulsivity which refers to the ability to prevent oneself from engaging in behaviors that are inappropriate for the situational context or require careful deliberation) are typically seen (Grillon et al., 2017). Moreover, research has found that presence of clinical anxiety may have a protective effect against response inhibition difficulties in ADHD (Maric et al., 2018; Rodríguez et al., 2014). When impulsivity is seen in clinical anxiety, it typically occurs within the context of negative affect (e.g., Pawluk & Koerner, 2013), whereas in ADHD impulsivity is not affect-dependent. As such, including excessive talking within the total score on Impulsivity subscale ASRS-v1.1 may further flag this discriminating factor, and increase accuracy of the screener.

An alternative explanation is that the *excessive talking* lacks utility in screening for ADHD altogether, as Kooij et al. (2005) found that this item had low loadings onto both Hyperactivity (.35) and Impulsivity (.33) in a three-factor model of ADHD in which items were allowed to cross-load. Proctor and Prevatt (2009) also found a low loading for this item (.34) in a three-factor model using informant-rated symptoms. Further investigation of how this item performs in various psychiatric populations is warranted to determine whether it should be reconceptualized as a symptom of Impulsivity or removed from the ASRS-v1.1 altogether.

### Discriminant Validity and Symptom Overlap

The ASRS-v1.1 total score demonstrated fair to good discriminant validity against measures of GAD and OCD, and good to excellent discriminant validity against the PD and social anxiety disorder measures. Discrimination against distress as measured by the DASS-21 was variable, depending on the relationship between the specific ADHD factor and distress subscale that was tested. There were moderate correlations between the Hyperactivity factor and DASS-21 Stress subscale, which is expected given similar language used for some items across the two measures (e.g., items asking about difficulty relaxing or winding in both). Overlap in item content may also explain the moderate correlation between the Inattention factor and DASS-21 Depression subscale (e.g., items related to delaying or avoiding task initiation are in both). These findings highlight the importance of clinicians considering the impact of stress and depression in clients with clinical anxiety who are endorsing ADHD symptoms. For example, clinicians should inquire about the mechanism underlying delaying or avoiding task initiation (e.g., lack of motivation related to depression or occurs independently of mood) as well as the onset (e.g., is this an ongoing problem they have always had, suggestive of ADHD, or has it varied with symptoms of anxiety/depression, suggestive of anxiety manifestation rather than ADHD).

Moreover, the specific factor of Impulsivity demonstrated the strongest discrimination of all three factors against all measures of anxiety or related disorders and distress. As discussed earlier, this factor may be particularly helpful when screening for ADHD in an anxiety or related disorders population, in which excessive response inhibition is more common than impulsivity. However, Hyperactivity demonstrated the poorest discriminant validity of the three factors. The ADHD item *having difficulty unwinding or relaxing* (item within the factor of Hyperactivity) demonstrated poor specificity with measures of generalized anxiety. This symptom is highly consistent with a typical GAD presentation, in which patients report worrying often about negative outcomes, even when things are going well. Changing this item to *having difficulty unwinding or relaxing, even in the absence of worries or anxiety*, may improve the specificity of the Hyperactivity factor when screening for ADHD in an anxiety or related disorders population. Alternatively, removing it from the screener altogether may be of benefit, as this item is not represented in the *DSM-IV*, *DSM-5*, or the most updated *Diagnostic and Statistical Manual of Mental Disorders* (5th ed., text rev.; *DSM-5-TR*; APA, 2022). Instead, this item could be replaced by one representing the ADHD symptom *often unable to play or take part in leisure activities quietly*, which not currently present in the ASRS-v.1.1 (APA, 2022). Interestingly, a study by Ustun et al. (2017) used a machine-learning algorithm to optimize the ASRS-v1.1 and included *difficulty unwinding* in their final six-item iteration of an optimal screener. They found high specificity for their screener within a general community-based sample and a clinical sample of individuals with ADHD (Ustun et al., 2017). However, whether this screener would demonstrate similarly high specificity in a clinical anxiety population has yet to be investigated.

The item *feeling overly active and compelled to do things, as if driven by a motor* loaded more significantly onto the OCD factor than the ADHD factor. These symptoms may be interpreted by patients as analogous to an urge to engage in a compulsion, which is a core symptom of OCD. This item may have improved specificity if reworded to delineate ADHD as the mechanism underlying the symptom rather than OCD (i.e., *feeling overly active, as if driven by a motor, even in the absence of compulsions or other anxiety symptoms*). Removal of the term *compelled* may reduce erroneous capture of compulsive symptoms in this clinical population.

Also, some items on the OCI-R subscales for neutralizing and obsessing loaded more strongly and significantly onto the ADHD factor than their respective OCD factor. These subscales capture symptoms that may occur due to poor motoric (e.g., neutralizing with compulsions) and cognitive inhibition (e.g., obsessions) seen in OCD. Research shows that poor response inhibition is characteristic of both OCD (Penadés et al., 2007; van der Linden et al., 2005) and ADHD (Alderson et al., 2007; Rubia et al., 2011; Sebastian et al., 2012). Interestingly, these impairments may have similar neurological underpinnings (for a review, see van Velzen et al., 2014). Similarly, hoarding items loaded strongly onto the ADHD factor. These loadings may also represent true comorbidity in our sample rather than erroneous loadings, as hoarding has been previously linked with the Inattention factor of ADHD (e.g., Morein-Zamir et al., 2021) and in those with OCD, hoarding behavior is associated with a 10-fold elevated risk of ADHD (Sheppard et al., 2010).

Taken together, these results suggest that despite some symptom overlap between ADHD and anxiety or related disorders, the ASRS-v1.1 is structurally valid and has fair discriminant validity, as items mostly loaded onto their ADHD factor even when pooled with anxiety or related disorder measures. However, there are several clinical implications of the poor discriminant properties of specific items within the Hyperactivity factor of the ASRS-v1.1. When screening for ADHD within an anxiety or related disorder population, it is recommended that clinicians avoid assuming Criterion A (e.g., Combined or Predominantly hyperactive/impulsive presentation) for ADHD is met unless the five symptoms reported are independent of anxiety symptoms. This is particularly critical for symptoms within the Hyperactivity factor (e.g., difficulty unwinding, feeling overly compelled to do things), in which it is recommended that clinicians investigate the mechanisms underlying reported symptoms. To differentiate between ADHD and clinical anxiety, assessors may benefit from specifically asking patients whether restlessness, problems relaxing, and feeling driven by a motor are still present in the absence of clinical anxiety symptoms or prior to onset of an anxiety or related disorder.

## Considerations, Limitations, and Future Directions

It is important to note that adult ADHD was not assessed in our sample, rather all participants completed a battery of questionnaires including the ASRS-v1.1 at clinic intake. Our sample had prevalence rates of reported diagnoses of ADHD more comparable to previously published rates in the general population than in psychiatric populations with anxiety or related disorders (Kessler et al., 2006; Van Ameringen et al., 2011). Despite this limitation, it has been found that ADHD is grossly underdiagnosed in adults with anxiety or related disorders (Pehlivanidis et al., 2014; Van Ameringen et al., 2011), often going undetected particularly in females and those with more inattentive presentations (Quinn & Wigal, 2004, see Dray et al., 2006; Quinn & Madhoo, 2014, for a review). Considering that our sample was 66% female, it is possible that some ADHD diagnoses were undetected or unreported. However, it is unlikely, based on previous prevalence rates that 41.3% of our sample had undetected ADHD (i.e., based on meeting threshold for Criterion A of ADHD as measured by the ASRS-v1.1). This suggests that the ASRS-v1.1 was capturing some non-ADHD symptoms and is thus susceptible to overinflated reporting of ADHD symptoms. While the ASRS-v1.1 has been validated for use in a variety of psychiatric populations (Carlucci et al., 2017; Dunlop et al., 2018, van de Glind et al., 2013) and we found moderate correlations between total scores on the ASRS-v1.1 and anxiety disorder-measures at most, it is still possible that this measure captured symptoms of other psychopathologies (e.g., cognitive impact of severe depression or chronic substance use; perfectionistic standards for cognition) in our sample rather than true ADHD symptoms. Future research with comprehensive assessment of ADHD is necessary to examine the specificity and sensitivity of the ASRS-v1.1 specifically in adult populations with anxiety or related disorders. Finally, investigating the model fits derived from the ASRS-v1.1 in comparison to other widely used screeners, such as the Conners’ Adult ADHD Rating Scale (CAARS; Conners et al., 1999), is essential to establish whether these models are replicated, and which screener is optimal for populations with clinical anxiety.

In addition, impairment caused by ADHD may extend into domains beyond Inattention, Hyperactivity, and Impulsivity such as emotional regulation and executive function (Hirsch et al., 2018; Surman et al., 2011). For example, when Westerlund et al. (2009) examined the factor structure of ADHD symptoms in a sample of children and included items related to emotional lability, they found a two-factor solution of traditional ADHD symptoms (e.g., Inattention, Hyperactivity, and Impulsivity) representing one factor and emotional lability representing the other factor best fit the data. Whether the three-factor model solution (Inattention, Hyperactivity-*r*, and Impulsivity-*r*) demonstrates superior fit in adult populations when other symptom domains such as emotional dysregulation are considered should be investigated. Moreover, previous research has indicated a discrepancy in factor structure of ADHD symptoms, depending on the informant (self or parent) in college students (Glutting et al., 2005). Given this finding, a comparison of model fits across multiple reporters may be useful, particularly in a clinical anxiety population in which respondents with perfectionistic perspectives on their cognitive abilities may over endorse symptoms such as difficulties concentrating. Finally, our sample was homogeneous in that it consisted of primarily White, educated individuals. It is known that racialized groups tend to be underdiagnosed with both ADHD and anxiety or related disorders alike (Shi et al., 2021; Vanderminden & Esala, 2019). It is necessary to examine the factor structure of ADHD in more diverse groups to understand whether there are differences in symptom structure of the ASRS-v1.1 across different populations with clinical anxiety.

## Conclusion

Given the increasing demands for mental health care and high rates of comorbidity between ADHD and anxiety or related disorders, tools that allow for routine, time-sensitive, and resource-effective screening of ADHD among clinicians treating adult anxiety are essential. The total score of the ASRS-v1.1 appears to pass the structural test of utility as a screening tool to detect potential ADHD in adults with principal anxiety or related disorders, but poor specificity of the Hyperactivity factor against some clinical anxiety symptoms necessitates more scrutiny of positive screens. Refinement or removal of non-specific items on the ASRS-v1.1 may better delineate between anxiety and ADHD, helping clinicians detect ADHD symptoms in adults with clinical anxiety to aid in treatment planning and decisions.

## Supplemental Material

sj-docx-1-asm-10.1177_10731911231225190 – Supplemental material for Are We Measuring ADHD or Anxiety? Examining the Factor Structure and Discriminant Validity of the Adult ADHD Self-Report Scale in an Adult Anxiety Disorder Population Supplemental material, sj-docx-1-asm-10.1177_10731911231225190 for Are We Measuring ADHD or Anxiety? Examining the Factor Structure and Discriminant Validity of the Adult ADHD Self-Report Scale in an Adult Anxiety Disorder Population by Arij Alarachi, Colleen Merrifield, Karen Rowa and Randi E. McCabe in Assessment
