# Supplementary material for: Are We Measuring ADHD or Anxiety? Examining the Factor Structure and Discriminant Validity of the Adult ADHD Self-Report Scale in an Adult Anxiety Disorder Population
Source: Assessment. 2024 Jan 30;31(7):1508–24. doi: 10.1177/10731911231225190 (PMC11409565; doi:10.1177/10731911231225190)
Supplement: sj-docx-1-asm-10.1177_10731911231225190 – Supplemental material for Are We Measuring ADHD or Anxiety? Examining the Factor Structure and Discriminant Validity of the Adult ADHD Self-Report Scale in an Adult Anxiety Disorder Population [file sj-docx-1-asm-10.1177_10731911231225190.docx]

**Are we measuring ADHD or anxiety? Examining the factor structure and discriminant validity of the Adult ADHD Self-Report Scale in an adult anxiety disorder population**

**Supplementary Materials**

**
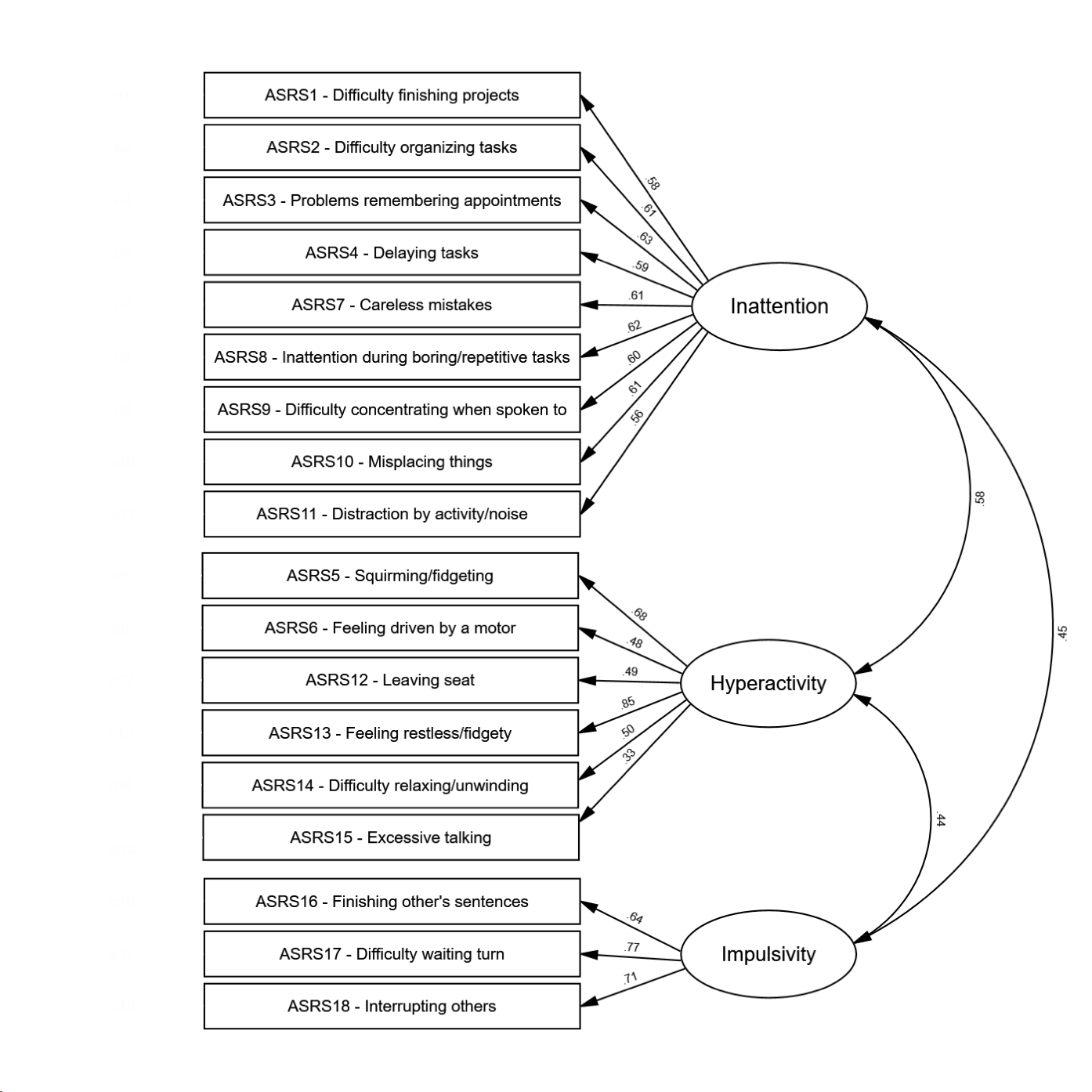
**

*Figure S1.* First-order correlated model of ASRS-v1.1 items with specific factors of Inattention, Hyperactivity, and Impulsivity. All factor loadings shown were significant (*p* < .001).

Table S1

*Standardized Regression Weights in the First-Order Three-Factor Model (Inattention, Hyperactivity-r, and Impulsivity-r)*

| Item | Specific Factor | Estimate | |  |  |
| --- | --- | --- | --- | --- | --- |
| ASRS1 | INATT | | **.58*** | | |
| ASRS2 | INATT | | **.61*** | |  |
| ASRS3 | INATT | | **.63*** | |  |
| ASRS4 | INATT | | **.59*** | |  |
| ASRS7 | INATT | | **.61*** | |  |
| ASRS8 | INATT | | **.62*** | |  |
| ASRS9 | INATT | | **.60*** | |  |
| ASRS10 | INATT | | **.61*** | |  |
| ASRS11 | INATT | | **.56*** | |  |
| ASRS5 | HYPER-R | | **.69*** | |  |
| ASRS6 | HYPER-R | | **.46*** | |  |
| ASRS12 | HYPER-R | | **.47*** | |  |
| ASRS13 | HYPER-R | | **.87*** | |  |
| ASRS14 | HYPER-R | | **.50*** | |  |
| ASRS16 | IMPUL-R | | **.70*** | |  |
| ASRS17 | IMPUL-R | | **.74*** | |  |
| ASRS18 | IMPUL-R | | **.71*** | |  |
| ASRS15 | IMPUL-R | | **.59*** | |  |

*Note*. Values with an asterisk indicate significance at *p* < .001 and in boldface indicate a stable factor loading (e.g., at least |.30|)

Table S2

*Item-Item Correlations in the First-Order Three-Factor Model (Inattention, Hyperactivity-r, and Impulsivity-r)*

| ASRS Item | 15 | 16 | 17 | 18 | 5 | 6 | 12 | 13 | 14 | 1 | 2 | 3 | 4 | 7 | 8 | 9 | 10 | 11 |
| --- | --- | --- | --- | --- | --- | --- | --- | --- | --- | --- | --- | --- | --- | --- | --- | --- | --- | --- |
| 15 | 1 | — | — | — | — | — | — | — | — | — | — | — | — | — | — | — | — | — |
| 16 | .395 | 1 | — | — | — | — | — | — | — | — | — | — | — | — | — | — | — | — |
| 17 | .439 | .497 | 1 | — | — | — | — | — | — | — | — | — | — | — | — | — | — | — |
| 18 | .419 | .475 | .528 | 1 | — | — | — | — | — | — | — | — | — | — | — | — | — | — |
| 5 | .165 | .187 | .207 | .198 | 1 | — | — | — | — | — | — | — | — | — | — | — | — | — |
| 6 | .11 | .124 | .138 | .132 | .321 | 1 | — | — | — | — | — | — | — | — | — | — | — | — |
| 12 | .112 | .126 | .14 | .134 | .327 | .217 | 1 | — | — | — | — | — | — | — | — | — | — | — |
| 13 | .206 | .233 | .259 | .248 | .603 | .402 | .409 | 1 | — | — | — | — | — | — | — | — | — | — |
| 14 | .117 | .133 | .148 | .141 | .343 | .229 | .233 | .43 | 1 | — | — | — | — | — | — | — | — | — |
| 1 | .155 | .176 | .195 | .186 | .226 | .151 | .153 | .283 | .161 | 1 | — | — | — | — | — | — | — | — |
| 2 | .163 | .185 | .205 | .196 | .238 | .159 | .161 | .298 | .17 | .358 | 1 | — | — | — | — | — | — | — |
| 3 | .166 | .189 | .209 | .2 | .243 | .162 | .165 | .304 | .173 | .366 | .385 | 1 | — | — | — | — | — | — |
| 4 | .157 | .177 | .197 | .188 | .229 | .152 | .155 | .286 | .163 | .344 | .362 | .37 | 1 | — | — | — | — | — |
| 7 | .162 | .184 | .204 | .195 | .236 | .157 | .16 | .296 | .168 | .356 | .375 | .383 | .36 | 1 | — | — | — | — |
| 8 | .163 | .185 | .206 | .196 | .238 | .159 | .162 | .298 | .17 | .359 | .378 | .386 | .363 | .376 | 1 | — | — | — |
| 9 | .16 | .181 | .201 | .192 | .233 | .155 | .158 | .292 | .166 | .351 | .37 | .377 | .355 | .367 | .37 | 1 | — | — |
| 10 | .163 | .184 | .205 | .196 | .238 | .158 | .161 | .297 | .169 | .358 | .377 | .384 | .362 | .374 | .377 | .369 | 1 | — |
| 11 | .148 | .168 | .186 | .178 | .216 | .144 | .146 | .27 | .154 | .326 | .343 | .35 | .329 | .341 | .343 | .336 | .342 | 1 |
